# PET-based assessment of coronary microvascular dysfunction and its relation to plaque burden and vascular remodeling: integration with microvascular resistance reserve

**DOI:** 10.1007/s00259-026-08054-3

**Published:** 2026-07-06

**Authors:** Yipu Ding, Roel Hoek, Putri Annisa Kamila, Ibrahim Danad, Nick S. Nurmohamed, Ruurt A. Jukema, Pieter G. Raijmakers, Roel S. Driessen, Pepijn A. van Diemen, Andrew D. Choi, Juhani Knuuti, Paul Knaapen, Hongbin Liu, Jeroen J. Bax, Alexander van Rosendael, on the behalf of the PACIFIC-1 Investigators., Roel S. Driessen, Pepijn van Diemen, Ruurt Jukema, Lilian Meijboom, Juhani Knuuti, Antti Saraste, Pieter G. Raijmakers, Paul Knaapen, Ibrahim Danad

**Affiliations:** 1 Department of Cardiology, Leiden University Medical Center, Leiden, The Netherlands; 2 School of Medicine, Nankai University, Tianjin, China; 3 Department of Cardiology, Amsterdam UMC, Vrije Universiteit Amsterdam, Amsterdam, The Netherlands; 4 Faculty of Medicine, Universitas Brawijaya, Malang, Indonesia; 5 Department of Vascular Medicine, Amsterdam UMC, University of Amsterdam, Amsterdam, The Netherlands; 6 Department of Radiology and Nuclear Medicine, Amsterdam UMC, Vrije Universiteit, Amsterdam, the Netherlands; 7 Division of Cardiology, The George Washington University School of Medicine, Washington, DC USA; 8 Turku University Hospital and University of Turku, Turku, Finland; 9 University Medical Center Utrecht, Utrecht, The Netherlands

**Keywords:** Coronary microvascular dysfunction, Coronary artery disease, Microvascular resistance reserve, Vascular remodeling, Plaque quantification

## Abstract

**Background:**

Coronary microvascular dysfunction (CMD) contributes to myocardial ischemia in patients with angina and non-obstructive coronary artery disease. Quantitative positron emission tomography (PET) allows noninvasive assessment of myocardial blood flow and identification of CMD.

**Objectives:**

To investigate the association between PET-assessed coronary microvascular dysfunction (CMD), coronary plaque burden, and vascular remodeling, integrating microvascular resistance reserve (MRR).

**Methods:**

This post hoc analysis of the PACIFIC-1 trial included coronary arteries from symptomatic patients with suspected stable CAD who underwent [^15^O]water PET, coronary CT angiography (CTA), and invasive fractional flow reserve (FFR). Coronary arteries were categorized into three groups: non-ischemic vessels (normal FFR and hyperemic myocardial blood flow [hMBF], *n* = 352), CMD vessels (normal FFR but impaired hMBF, *n* = 88), and epicardial flow-limiting vessels (abnormal FFR, *n* = 159). MRR was calculated by integrating PET-derived coronary flow reserve with invasive FFR. AI-based CTA quantified plaque burden and remodeling.

**Results:**

Diameter stenosis and plaque burden increased progressively from non-ischemic to CMD to epicardial flow-limiting vessels (all *P* < 0.05). CMD vessels demonstrated the largest lumen volume (583.75 mm^3^ [IQR 337.02–754.30]) and lumen area (4.63 mm^2^ [IQR 3.77–6.67]) among the three groups (both *P* < 0.05). In non-ischemic vessels, noncalcified plaque burden was associated with worse microvascular function detected by MRR (B = -0.12, 95%CI [-0.21, -0.04], *P* = 0.006). In CMD vessels, remodeling index was independently associated with higher MRR after adjustment (B = 0.10, 95%CI [0.05, 0.15], *P* < 0.001).

**Conclusions:**

CMD is associated with an intermediate atherosclerotic burden and paradoxical lumen enlargement, representing a distinct functional-structural phenotype defined by PET-derived microvascular dysfunction and CT-based plaque characteristics.

**Clinical Trial Registration:**

URL: https://www.clinicaltrials.gov; Unique identifier: NCT01521468.

**Graphical Abstract:**

Abbreviations: CMD, coronary microvascular dysfunction; FFR, fractional flow reserve; MBF, myocardial blood flow; MRR, microvascular resistance reserve; PET, positron emission tomography-computed tomography.

**Figure:**
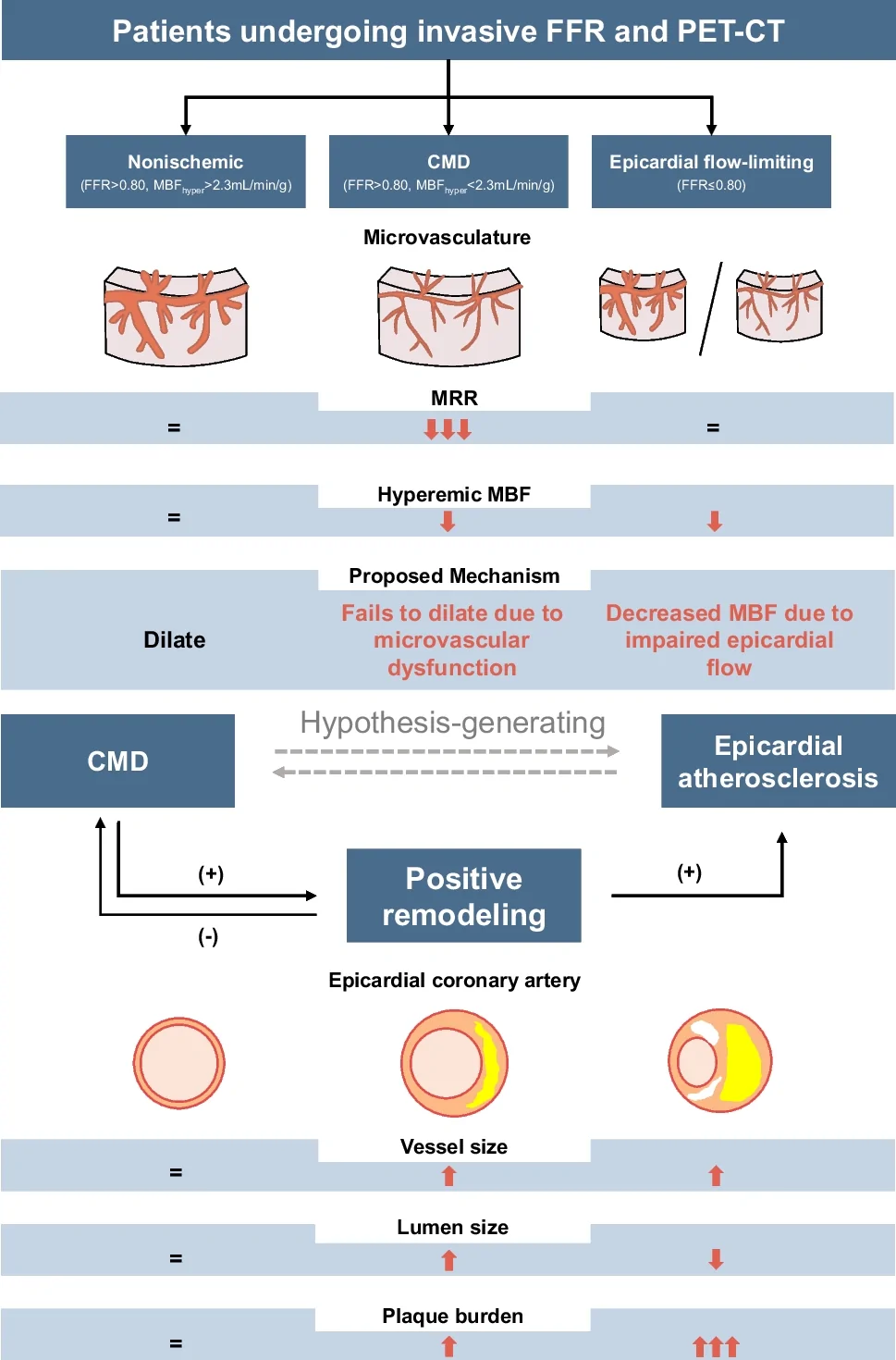
Graphical abstract. Abbreviations: CMD, coronary microvascular dysfunction; FFR, fractional flow reserve; MBF, myocardial blood flow; MRR, microvascular resistance reserve; PET, positron emission tomography-computed tomography

**Supplementary Information:**

The online version contains supplementary material available at https://doi.org/10.1007/s00259-026-08054-3.

## Introduction

It is now well established that chronic coronary syndromes (CCS) with ischemia frequently occur in the absence of flow-limiting epicardial stenosis. Approximately 50% of patients referred for coronary angiography with angina do not exhibit obstructive coronary artery disease (CAD) [1–3]. This clinical scenario, now increasingly recognized as angina with non-obstructive coronary arteries (ANOCA), has received growing attention in the recent European Society of Cardiology guideline on CCS, which emphasize the need for its proper diagnosis and management [4].

Coronary microvascular disease (CMD) represents one of the key mechanisms underlying ANOCA, contributing to myocardial ischemia and adverse cardiovascular outcomes despite angiographically normal or near-normal coronary arteries [5, 6]. Although CMD has traditionally been viewed as a distinct pathological entity separate from epicardial coronary artery atherosclerosis, emerging evidence suggests that the boundary between these two processes may be more complex than previously considered [7]. Recent studies indicate a potential interplay between early epicardial plaque formation and microvascular dysfunction, pointing to a pro-atherogenic environment within the epicardial vessels of patients with CMD [8].

Advances in quantitative PET imaging, combined with invasive physiological indices, have enabled comprehensive assessment of both macrovascular and microvascular coronary function [10–12]. In this framework, PET provides the quantitative basis for myocardial blood flow assessment, while microvascular resistance reserve (MRR) extends this approach by integrating invasive pressure measurements into an epicardial flow-independent index of microvascular function [13].

In this study, we sought to investigate the relationship between epicardial atherosclerotic characteristics by quantitative coronary CT and CMD by integrating PET and invasive fractional flow reserve (FFR). Our aim was to improve understanding of the epicardial vascular responses associated with microvascular dysfunction and to explore their potential implications for the early development of CAD.

## Methods

### Study population

The current study is a post hoc analysis of the Prospective Comparison of Cardiac PET/CT, SPECT/CT Perfusion Imaging and CT Coronary Angiography With Invasive Coronary Angiography (PACIFIC-1) trial. The PACIFIC-1 trial was a prospective controlled clinical single-center trial conducted from January, 2012, to October, 2014, at the Free University Medical Center, Amsterdam, the Netherlands (NCT01521468) [14]. All participants (*n* = 208), with suspected obstructive CAD, completed a 2-week protocol including [^15^O]water PET and coronary computed tomography angiography (CCTA) prior to invasive coronary angiography (ICA) in combination with routine three-vessel invasive FFR examination. All index tests were interpreted blindly by core laboratories. The study protocol was approved by the Medical Ethics Committee of the Free University Medical Center and all participants provided written informed consent.

### PET procedure

PET scans were performed using a hybrid PET/CT equipment (Philips Gemini TF 64, Philips Healthcare, Best, the Netherlands). The scan procedure involved a dynamic 6 min scanning protocol that initiated simultaneously with an injection of 370 MBq [^15^O]water PET during resting and adenosine-induced hyperemic conditions (140 µg/kg/min). Low-dose CT scans were performed for attenuation correction. Parametric MBF images were created and quantitatively analyzed using Carimas software (Turku PET Centre, University of Turku and Turku University Hospital, Turku, Finland). MBF was expressed in mL/min/g of perfusable tissue and analyzed according to the 17 segments of the left ventricle as per the American Heart Association model with standardized allocation of segments to the three vascular territories [15]. Regional hyperemic myocardial blood flow (hMBF) was defined as mean hMBF of the entire vascular territory in the absence of a perfusion defect or as the mean hMBF of the perfusion defect (≥ 2 adjacent segments with a hMBF ≤ 2.3 mL/min/g) when present [16]. Coronary flow reserve (CFR) was defined as the ratio of hMBF to resting MBF (rMBF). Coronary arteries were categorized into three groups: non-ischemic vessels (normal FFR and normal hMBF), CMD vessels (normal FFR but impaired hMBF), and epicardial flow-limiting vessels (abnormal FFR). In this framework, CMD vessels were defined as non-flow-limiting epicardial vessels subtending myocardial territories with PET evidence of impaired hyperemic perfusion.

### ICA and physiological assessments

ICA was performed according to standard clinical protocols [14]. All major coronary arteries (> 2 mm) were routinely interrogated by FFR, irrespective of stenosis severity and imaging results. To induce maximal coronary hyperemia, adenosine was administered intracoronary as a 150 μg bolus or intravenously (140 μg/kg/min). FFR was calculated as the ratio of mean distal intracoronary to aortic guiding pressure during hyperemia [17]. An FFR ≤ 0.80 was graded as abnormal. Vessels in which FFR was not required due to stenosis severity were considered as ischemic as well. MRR was derived based on the framework by De Bruyne et al. by combining invasive FFR measurements and noninvasive PET flow measurements [13]. The formula used in the current analysis is a quotient of CFR and FFR with the correction for the impact of hemodynamics, as follows:

$$\mathrm{MRR}=(\mathrm{CFR}/\mathrm{FFR})\times{\mathrm P}_{\mathrm a,\mathrm{rest}}/{\mathrm P}_{\mathrm a,\mathrm{hyper}},$$

where CFR indicates PET-derived CFR, FFR indicates pressure-wire derived FFR, and P_a,rest_ and P_a,hyper_ indicate mean aortic pressure during non-hyperemic and maximal hyperemic PET, respectively. Based on prior research [18, 19], we adopted a prognostically significant threshold of MRR ≤ 3.0 as a second marker for CMD.

### Coronary CTA acquisition

CT scans were performed using a 256-slice CT scanner (Philips Brilliance iCT, Philips Healthcare, Best, the Netherlands) with a tube current between 200 and 360 mAs at 120 kV. Respective CT parameters for the 256-slice entailed a section collimation of 128 × 0.625 mm and a gantry rotation time of 270 ms. Each patient received 800 mcg of sublingual nitroglycerine immediately prior to CCTA. If necessary, metoprolol was given prior to CCTA by oral or intravenous administration. For visualization of the coronary artery lumen, a bolus of iobitridol (Xenetix 350) was injected intravenously followed immediately by a saline chaser. Prospective electrocardiogram gating between 72 and 78% of the R-R interval was performed to reduce radiation dose [14, 20].

### Artificial intelligence-guided analysis of quantitative computed tomography (AI-QCT) from coronary CTA

Coronary CTA scans were analyzed using the previously described AI-QCT algorithm [21, 22]. The quantitative coronary CTA evaluation was performed blinded to the clinical and outcomes data. This Food and Drug Administration (FDA)-cleared software uses a series of validated convolutional neural networks (3-dimensional U-Net and Visual Geometry Group [VGG] network variants) for image-quality assessment, coronary artery segmentation and labeling, lumen wall evaluation and vessel contour determination, and plaque characterization.

Total plaque volume (TPV) (mm^3^) was defined as the sum of all plaque volumes calculated for each coronary artery lesion among all vessels. Noncalcified plaque (NCP) was defined as a lesion with a CT density between 30 and 350 HU, and calcified plaque (CP) above 350 HU [23]. The percentage of atheroma volume (PAV) was calculated by dividing plaque volume (PV)/vessel volume × 100%. Positive remodeling was defined by a remodeling index of ≥ 1.1, which was calculated by the vessel size at the site of the maximal remodeling divided by the vessel size of the reference site [24].

### Statistical analysis

Continuous variables were described as mean ± standard deviation or median (interquartile range), and compared between the groups using either 2 sample t-test or nonparametric Wilcoxon rank sum test. Categorical variables were summarized as counts (percentages) and compared using the Pearson chi-square test or Fisher exact test if cell frequencies were insufficient. Bonferroni adjustment was applied to account for multiple testing.

**Fig. 1:**
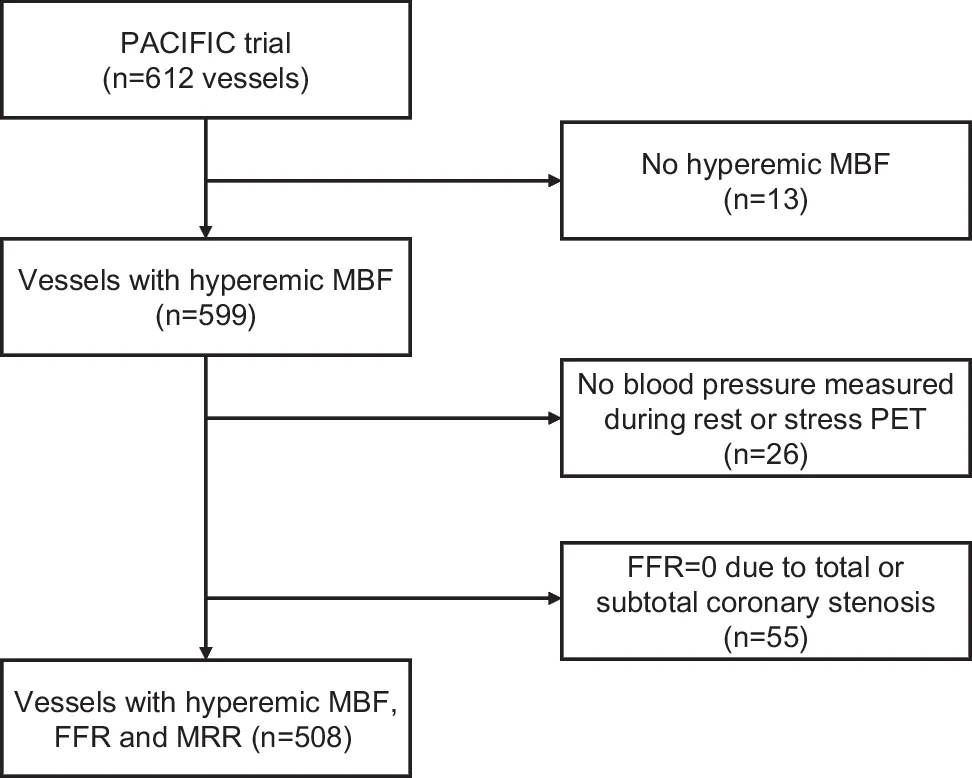
Flow chart. Abbreviations: FFR, fractional flow reserve; MBF, myocardial blood flow; MRR, microvascular resistance reserve; PET, positron emission tomography

All analyses were performed at the vessel level. For the primary analyses, 599 vessels with complete hMBF data were included. A subset of 508 vessels, with complete data for FFR, hMBF, and MRR, was used for analyses involving MRR. A detailed flowchart outlining vessel inclusion and exclusion is presented in Fig. 1. To correct for cardiovascular risk factors and for multiple vessels within a patient, generalized estimating equations (GEEs) were used to assess the relation between risk factors and vessel-specific MRR. A two-sided *P* < 0.05 was considered statistically significant. Significant predictors of vessel-specific MRR in the univariable analysis were included into the multivariable model. Sensitivity analyses were performed adjusting for alternative plaque composition parameters and for coronary vessel type. Statistical analysis was performed using SPSS version 22.0 (SPSS, IL, USA), GraphPad Prism 8.0 (GraphPad Software, USA), MedCalc 19.0.4 (MedCalc Software, Belgium) and R version 3.6.3 (R Foundation for Statistical Computing, Vienna, Austria).

## Results

### Clinical characteristics

As shown in Table 1, a total of 599 vessels with complete FFR and hMBF data were included, comprising 352 (58.8%) non-ischemic vessels, 88 (14.7%) with CMD, and 159 (26.5%) with epicardial flow-limiting disease. A higher prevalence of male sex, diabetes, hyperlipidemia and smoking history was found in CMD vessels and epicardial flow-limiting vessels compared with non-ischemic vessels (all *P* < 0.05). Medication use was generally comparable across groups, with the exception of significantly higher use of aspirin (epicardial flow-limiting: 93.1% [148/159] vs. non-ischemic: 84.7% [298/352]; *P* = 0.009) and statin (epicardial flow-limiting: 86.2% [137/159] vs. non-ischemic: 73.0% [257/352]; *P* = 0.001) in the epicardial flow-limiting group.

**Table 1:** Vessel-level clinical characteristics

Characteristics	Overall (*n* = 599)	Non-ischemic (*n* = 352)	CMD (*n* = 88)	Epicardial flow-limiting (*n* = 159)	*P*-value
Age, years	59.00 [52.00, 64.00]	58.00 [51.00, 64.00]	60.00 [52.00, 66.00]	60.00 [53.00, 65.00]	0.580
Male, %	376 (62.8)	164 (46.6)	74 (84.1)	138 (86.8)	< 0.001^ac^
BMI, kg/m^2^	26.84 [24.36, 29.34]	26.54 [23.74, 29.05]	26.70 [24.62, 29.05]	27.17 [24.90, 29.64]	0.134^a^
Diabetes mellitus, %	94 (15.7)	44 (12.5)	17 (19.3)	33 (20.8)	0.036
Hypertension, %	281 (46.9)	166 (47.2)	41 (46.6)	74 (46.5)	0.990
Hyperlipidemia, %	237 (39.6)	121 (34.4)	36 (40.9)	80 (50.3)	0.003^c^
Smoking history, %	294 (49.1)	152 (43.2)	52 (59.1)	90 (56.6)	0.002^ac^
Family history of CAD, %	305 (50.9)	183 (52.0)	42 (47.7)	80 (50.3)	0.762
Symptoms, %					< 0.001^ac^
Typical angina	210 (35.1)	99 (28.1)	46 (52.3)	65 (40.9)	
Atypical angina	222 (37.1)	145 (41.2)	27 (30.7)	50 (31.4)	
Aspecific chest pain	167 (27.9)	108 (30.7)	15 (17.0)	44 (27.7)	
Medication					
Aspirin, %	525 (87.6)	298 (84.7)	79 (89.8)	148 (93.1)	0.022^c^
Statin, %	465 (77.6)	257 (73.0)	71 (80.7)	137 (86.2)	0.003^c^
ACEI/ARB, %	226 (37.7)	134 (38.1)	35 (39.8)	57 (35.8)	0.813
Beta blocker, %	394 (65.8)	219 (62.2)	62 (70.5)	113 (71.1)	0.090
CCB, %	177 (29.5)	109 (31.0)	18 (20.5)	50 (31.4)	0.128
Long-acting nitrates, %	64 (10.7)	44 (12.5)	9 (10.3)	11 (7.0)	0.180

Values are n (%) or median [IQR]

^a^ indicates a significant difference between non-ischemic vessels and CMD vessels (Bonferroni-adjusted *P* < 0.05)

^b^ indicates a significant difference between CMD vessels and epicardial flow-limiting vessels (Bonferroni-adjusted *P* < 0.05)

^c^ indicates a significant difference between non-ischemic vessels and epicardial flow-limiting vessels (Bonferroni-adjusted *P* < 0.05)

*ACEI/ARB* angiotensin converting enzyme inhibitor/angiotensin receptor blocker; *BMI* body mass index; *CAD* coronary artery disease; *CCB* calcium channel blocker; *CMD* coronary microvascular dysfunction

### Imaging characteristics

**Fig. 2:**
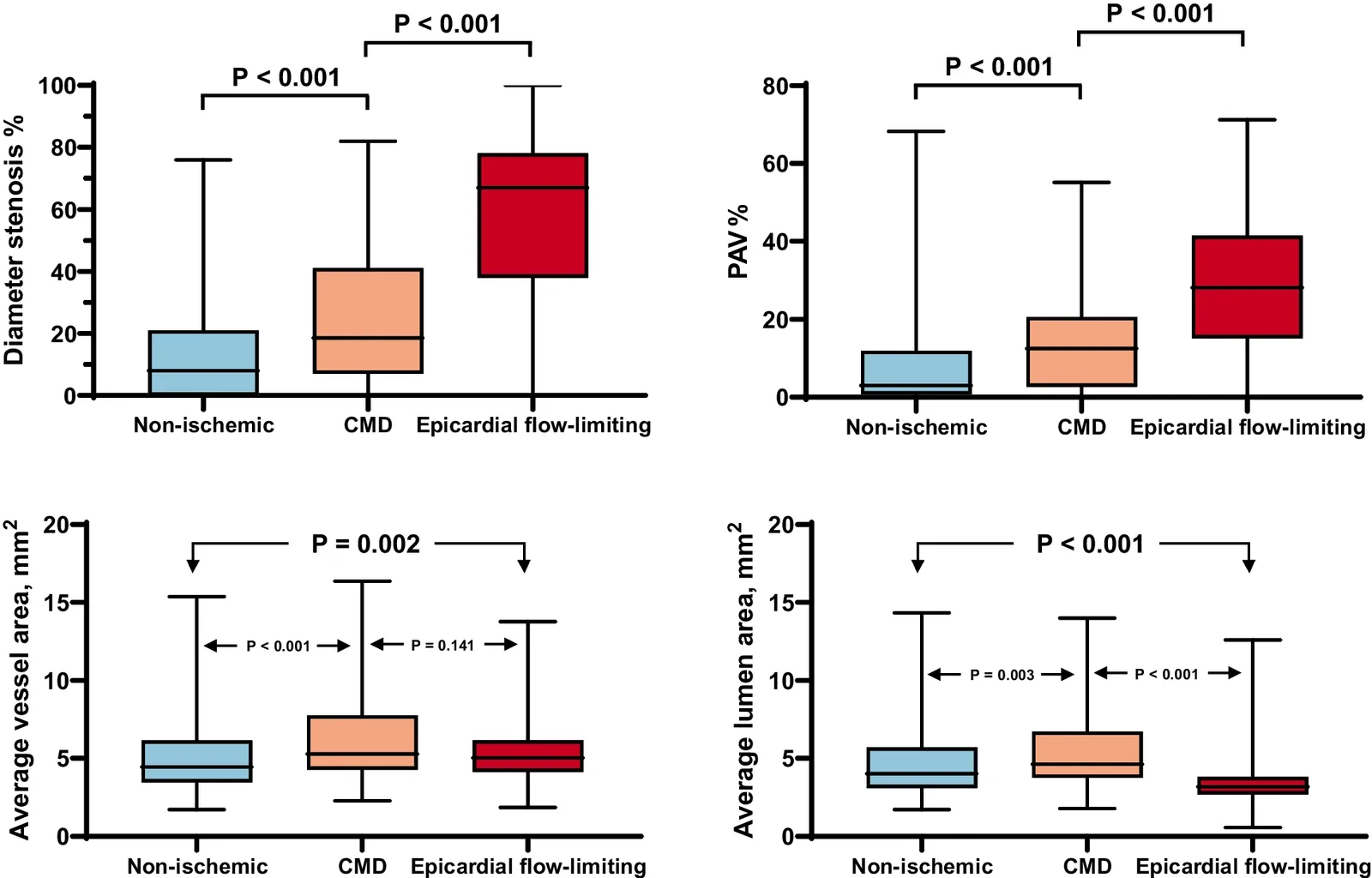
The comparison of stenosis severity, plaque burden and vessel size between the groups. The box indicates the interquartile range (25th to 75th percentiles), and the central line marks the median. The whiskers extend to the minimum and maximum values. Abbreviations: CMD, coronary microvascular dysfunction; PAV, percentage atheroma volume

Atherosclerotic burden increased progressively across non-ischemic, CMD, and epicardial flow-limiting vessels. This pattern was reflected in diameter stenosis (non-ischemic: 8.00%, CMD: 18.50%, epicardial flow-limiting: 67.00%; *P* < 0.001), plaque volume (non-ischemic: 12.60 mm^3^ [IQR 2.88–59.30], CMD: 50.40 mm^3^ [IQR 12.20–139.85], epicardial flow-limiting: 167.10 mm^3^ [IQR 80.05–269.65]; *P* < 0.001), and PAV (non-ischemic: 3.01% [IQR 0.77–11.89], CMD: 12.52% [IQR 3.06–20.52], epicardial flow-limiting: 28.17% [IQR 15.35–41.36]; *P* < 0.001) (Table 2 and Fig. 2).

**Table 2:** Vessel-level imaging characteristics

Characteristics	Overall (*n* = 599)	Non-ischemic (*n* = 352)	CMD (*n* = 88)	Epicardial flow-limiting (*n* = 159)	*P*-value
Baseline MBF	1.00 [0.90, 1.30]	1.10 [0.90, 1.30]	0.90 [0.80, 1.10]	0.90 [0.80, 1.10]	< 0.001^ac^
Hyperemic MBF	3.10 [2.00, 4.00]	3.80 [3.20, 4.40]	1.90 [1.67, 2.10]	1.80 [1.30, 2.10]	< 0.001^ac^
Diameter stenosis, %	17.00 [4.00, 50.00]	8.00 [0.00, 21.00]	18.50 [7.00, 41.00]	67.00 [38.50, 78.00]	< 0.001^abc^
Vessel volume, mm^3^	531.60 [359.00, 763.65]	486.40 [305.20, 685.45]	733.95 [379.25, 912.60]	599.50 [447.25, 775.85]	< 0.001^ac^
Lumen volume, mm^3^	436.90 [282.40, 626.55]	439.60 [279.78, 637.85]	583.75 [337.02, 754.30]	400.00 [273.90, 523.45]	< 0.001^ab^
Average vessel area, mm^2^	4.77 [3.70, 6.36]	4.45 [3.47, 6.15]	5.28 [4.28, 7.69]	5.02 [4.15, 6.16]	< 0.001^ac^
Average lumen area, mm^2^	3.77 [2.95, 5.43]	4.00 [3.12, 5.68]	4.63 [3.77, 6.67]	3.19 [2.71, 3.80]	< 0.001^abc^
Remodeling index	1.30 [1.20, 1.50]	1.20 [1.10, 1.40]	1.30 [1.20, 1.42]	1.50 [1.30, 1.60]	< 0.001^bc^
Positive remodeling, %	162 (27.0)	56 (15.9)	22 (25.0)	84 (52.8)	< 0.001^bc^
Fractional flow reserve	0.92 [0.78, 0.97]	0.95 [0.91, 0.99]	0.94 [0.89, 0.97]	0.70 [0.53, 0.76]	< 0.001^abc^
Plaque volume, mm^3^					
Total plaque	38.60 [6.45, 139.65]	12.60 [2.88, 59.30]	50.40 [12.20, 139.85]	167.10 [80.05, 269.65]	< 0.001^abc^
Calcified plaque	10.60 [0.00, 56.15]	0.45 [0.00, 22.25]	11.25 [0.00, 54.27]	57.70 [21.15, 128.90]	< 0.001^abc^
Non-calcified plaque	22.90 [5.20, 70.40]	8.90 [2.80, 32.28]	36.60 [11.30, 81.23]	81.80 [50.20, 122.75]	< 0.001^abc^
Percentage atheroma volume, %					
Total plaque	8.77 [1.46, 22.60]	3.01 [0.77, 11.89]	12.52 [3.06, 20.52]	28.17 [15.35, 41.36]	< 0.001^abc^
Calcified plaque	2.21 [0.00, 9.26]	0.07 [0.00, 4.56]	2.27 [0.00, 9.56]	10.17 [3.80, 22.70]	< 0.001^abc^
Non-calcified plaque	4.58 [1.35, 11.44]	2.24 [0.76, 6.30]	7.01 [2.17, 11.09]	13.53 [8.76, 20.76]	< 0.001^abc^

Values are n (%) or median [IQR]

^a^ indicates a significant difference between non-ischemic vessels and CMD vessels (Bonferroni-adjusted *P* < 0.05)

^b^ indicates a significant difference between CMD vessels and epicardial flow-limiting vessels (Bonferroni-adjusted *P* < 0.05)

^c^ indicates a significant difference between non-ischemic vessels and epicardial flow-limiting vessels (Bonferroni-adjusted *P* < 0.05)

*CMD* coronary microvascular dysfunction

CMD vessels had the largest lumen volume (583.75 mm^3^ [IQR 337.02–754.30]) and average lumen area (4.63 mm^2^ [IQR 3.77–6.67]) among the three groups (both *P* < 0.05). In contrast, vessel volume (CMD: 733.95 mm^3^ [IQR 379.25–912.60] vs. epicardial flow-limiting: 599.50 mm^3^ [IQR 447.25–775.85]; P = 0.263) and average vessel area (CMD: 5.28 mm^2^ [IQR 4.28–7.69] vs. epicardial flow-limiting: 5.02 mm^2^ [IQR 4.15–6.16]; P = 0.141) were similar between CMD and epicardial flow-limiting vessels, but both were significantly larger than in non-ischemic vessels (all *P* < 0.05) (Fig. 2). In accordance, the prevalence and extent of positive remodeling showed a stepwise increase across the three groups (Table 2).

### Distribution of MRR

**Fig. 3:**
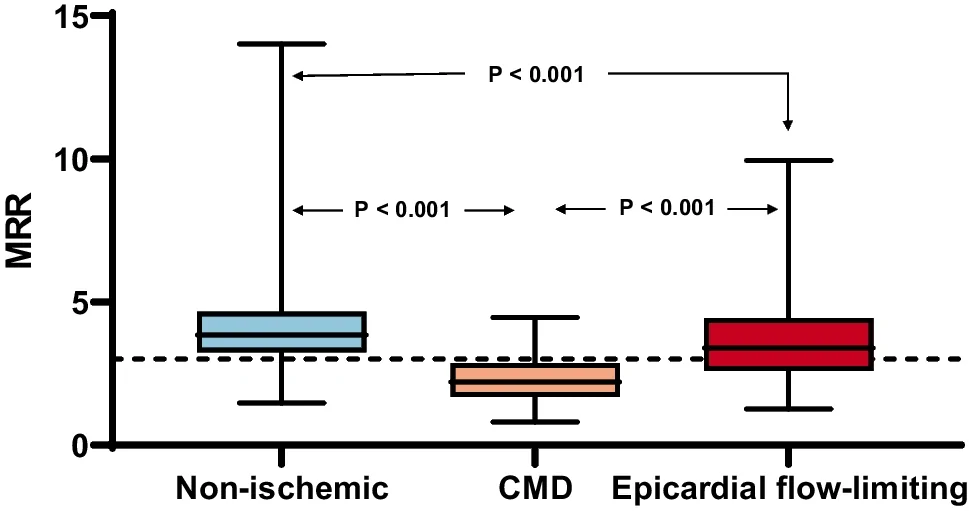
The comparison of MRR between the groups. The results were calculated in the 508 coronary vessels with full FFR data. The box indicates the interquartile range (25th to 75th percentiles), and the central line marks the median. The whiskers extend to the minimum and maximum values. The grey dashed line indicates an MRR of 3. Abbreviations: CMD, coronary microvascular dysfunction; MRR, microvascular resistance reserve

A total of 508 vessels with complete FFR, hMBF, and MRR data were included in the MRR-related analysis, comprising 329 (64.8%) non-ischemic vessels, 83 (16.3%) CMD vessels, and 96 (18.9%) epicardial flow-limiting vessels. As shown in Fig. 3, CMD vessels demonstrated a significantly lower MRR (2.22 [IQR 1.71–2.85]) compared with both non-ischemic vessels (3.86 [IQR 3.26–4.67]) and epicardial flow-limiting vessels (3.40 [IQR 2.62–4.43]) (*P* < 0.001 for both comparisons), with 79.5% of CMD vessels exhibiting an MRR ≤ 3.00.

### Predictors of MRR

In the CMD group (Table 3), univariable analysis showed that remodeling index (B = 0.12, 95% CI [0.07, 0.17], *P* < 0.001), total PAV, PAV-CP, and PAV-NCP were associated with MRR. After adjustment for PAV-NCP, higher remodeling index (per 10% increase) remained significantly associated with higher MRR (B = 0.10, 95% CI [0.05, 0.15], *P* < 0.001). Additional multivariable analyses adjusting separately for total PAV, PAV-CP, and vessel type are presented in Tables S3 and S4, and consistently demonstrated an independent association between higher remodeling index and higher MRR (all *P* < 0.05).

**Table 3:** Prediction of MRR in coronary microvascular dysfunction vessel**s**

Parameters	Univariable		Multivariable	
	B (95%CI)	*P*-value	B (95%CI)	*P*-value
Age, per 1 year increase	−0.02 (−0.04, 0.00)	0.114		
Male	0.33 (−0.09, 0.74)	0.121		
BMI, per 1 kg/m^2^ increase	0.02 (−0.03, 0.08)	0.426		
Diabetes mellitus	−0.13 (−0.62, 0.35)	0.593		
Hypertension	−0.17 (−0.58, 0.25)	0.424		
Hyperlipidemia	0.22 (−0.19, 0.62)	0.293		
Smoking history	0.04 (−0.36, 0.44)	0.851		
Family history of CAD	0.07 (−0.35, 0.49)	0.734		
Symptoms				
Typical angina	Reference			
Atypical angina	0.39 (−0.16, 0.94)	0.162		
Aspecific chest pain	−0.01 (−0.46, 0.45)	0.982		
Imaging characteristics				
Diameter stenosis, per 5% increase	0.02 (−0.01, 0.05)	0.119		
Diameter stenosis ≥ 50%	0.16 (−0.14, 0.45)	0.301		
Remodeling index, per 10% increase	0.12 (0.07, 0.17)	< 0.001	0.10 (0.05, 0.15)	< 0.001
Average lumen area, per 1 mm^2^ increase	−0.03 (−0.07, 0.00)	0.062		
Percentage atheroma volume, per 5% increase				
Total plaque	0.09 (0.03, 0.15)	0.002		
Calcified plaque	0.11 (0.02, 0.20)	0.016		
Non-calcified plaque	0.18 (0.03, 0.33)	0.021	0.10 (−0.04, 0.24)	0.151

*BMI* body mass index; *CAD* coronary artery disease; *CI* confidence interval

In the non-ischemic vessel group, only total PAV and PAV-NCP were significantly associated with MRR, both showing a negative correlation (Table S1). In the epicardial flow-limiting group, the presence of hypertension (B = −0.80, 95% CI [−1.36, −0.23], *P* = 0.006), remodeling index, and PAV-NCP were associated with lower MRR in univariable analysis. After adjustment, only hypertension remained independently associated with reduced MRR (B = −0.73, 95% CI [−1.26, −0.21], *P* = 0.007) (Table S2).

## Discussion

This study investigated coronary physiology and morphology across three groups of vessels stratified by [^15^O]water PET-derived hyperemic myocardial blood flow and invasive FFR. We found that vessels with coronary microvascular dysfunction exhibited an intermediate degree of atherosclerosis, greater than non-ischemic vessels but lower than flow-limiting epicardial disease. At the same time, CMD vessels demonstrated increased vessel and lumen size, consistent with positive remodeling. In contrast, vessels with flow-limiting disease showed comparable vessel size but the smallest lumen area, reflecting luminal encroachment. Within this framework, CMD may represent a distinct structural–functional phenotype characterized by the coexistence of microvascular dysfunction, plaque accumulation, and adaptive vascular remodeling.

### Potential mechanisms

Our data extend these observations by demonstrating that CMD vessels on CT exhibit a distinctive structural–functional profile. Among the three groups, CMD vessels had the largest lumen volume and area indexed by vessel length, indicating pronounced positive remodeling, and significantly greater atherosclerotic burden (higher diameter stenosis, plaque volume, and plaque area volume) compared to non-ischemic vessels [25–27]. This pattern suggests that CMD is not merely an indistinct intermediate but may reflect a phenotype in which microvascular dysfunction, epicardial plaque accumulation and compensatory arterial changes coexist.

At an earlier stage, even vessels classified as “non-ischemic” already showed an association between higher plaque burden and lower MRR (Table S1), consistent with previous studies linking early atherosclerosis to microvascular impairment [28, 29]. Interestingly, remodeling index was found related to higher MRR in CMD vessel, which might possibly indicate an adaptive stage during which the epicardial vessels can still positively remodel to maintain perfusion (around 12% PAV). We hypothesize that positive remodeling may temporarily mitigate microvascular impairment while simultaneously creating low endothelial shear stress (ESS), a hemodynamic environment known to promote plaque progression [30, 31]. This hypothesis is partially supported by Siasos et al. [8], who found that coronary segments from patients with non-obstructive CAD and abnormal microvascular function exhibited lower ESS than those with normal microvascular function. Although our definition of CMD differs, their results provide a relevant physiological context.

Importantly, the nearly normal or even slightly enlarged lumen size of CMD vessels observed in our study, a feature reported previously [25, 26, 32] but often overlooked, emphasizes that epicardial arterial compensation may already be present in vessels with microvascular dysfunction, even in the absence of flow-limiting stenosis. By analyzing a larger number of vessels, we were able to highlight this phenomenon, which has traditionally been attributed only to epicardial atherosclerosis [33]. In support of this, a previous study in patients with angina and non-obstructive CAD demonstrated a significant inverse correlation between lumen volume and minimal microvascular resistance [26], further reinforcing the idea that positive remodeling may occur in response to microvascular impairment.

### Microvascular resistance reserve

MRR, a robust metric reflecting microvascular vasodilatory capacity, was incorporated in our analysis as a key index of microvascular function. Previous studies have demonstrated that MRR is independently associated with major adverse cardiac events over a 5-year follow-up, regardless of the presence/absence of flow-limiting epicardial disease [18], and may serve as a valuable reference measure of coronary microcirculation [34]. Prior work from our team also established the utility of MRR in patients with suspected CAD and revealed systemic associations between plaque burden and microvascular function [35, 36]. However, the current study uncovered an interesting pattern of MRR change across different vessel types. In non-ischemic vessels, non-calcified plaque burden was associated with a decrease in MRR, whereas in CMD vessels, it was associated with increased MRR prior to adjustment for positive remodeling. This seemingly paradoxical observation once again highlights the potential compensatory role of positive remodeling, which may initially buffer microvascular dysfunction while inadvertently facilitating plaque progression. Nevertheless, given the observational nature of this study, the directionality and causality of these associations cannot be definitively established. Longitudinal studies will be required to clarify the temporal relationship between early atherosclerosis, positive remodeling, and microvascular dysfunction.

### Clinical implications

Patients with anginal symptoms but no angiographic evidence of obstructive coronary disease are often underdiagnosed and discharged without targeted treatment or follow-up, under the assumption of “normal” coronary arteries [28]. However, individuals with CMD, despite having predominantly non-obstructive CAD, already exhibit a median plaque volume of 50.40 mm^3^ or 12.5% plaque burden, corresponding to approximately the 60th percentile in men and the 80th percentile in women [37], levels that are themselves associated with an increased risk of future coronary events [28, 38, 39]. These patients therefore deserve closer clinical attention. In particular, non-obstructive vessels with a normal or even enlarged lumen but relatively high plaque burden, features suggestive of CMD and positive remodeling, may represent a critical window during which timely intervention could potentially slow or reverse atherosclerosis; however, this possibility requires confirmation in longitudinal studies. Our study highlights this potential interplay between myocardial microvascular dysfunction and early epicardial atherosclerosis, suggesting a potentially under-recognized association between early epicardial atherosclerotic changes and microvascular dysfunction that may have implications for earlier disease detection. This finding may help raise awareness among clinicians and patients regarding the importance of CMD as an early contributor to disease progression. Moreover, the integration of MRR in our study demonstrates its potential utility in future research. Combining PET-based microvascular assessments with noninvasive epicardial hemodynamic indices such as CT-derived FFR could offer a promising, fully noninvasive approach to CMD evaluation, paving the way for broader clinical application.

### Limitations

First, this study was a post hoc analysis of the single center PACIFIC study, which may introduce selection bias and limit generalizability. In addition, the use of [^15^O]water PET requires specialized infrastructure and expertise for handling ultra-short half-life tracers, potentially limiting broader clinical applicability of the findings. Furthermore, because PACIFIC-1 was originally designed to evaluate patients with suspected obstructive CAD rather than CMD or ANOCA specifically, women and patients with classical microvascular angina phenotypes were underrepresented in the current cohort, which may have introduced selection bias and reduced applicability to broader CMD populations. Second, endothelial function testing and invasive assessment of microvascular resistance were not available, restricting direct mechanistic interpretation. Third, as an observational study, the current analysis does not include clinical outcome data, limiting prognostic inferences. Fourth, due to the cross-sectional nature of the data, causal relationships between CMD, positive remodeling, and plaque progression could not be established; the current findings remain hypothesis-generating. Future serial studies are warranted to further confirm the hypothesis. Fifth, 55 coronary arteries with total or subtotal occlusion were excluded from the MRR analysis, which may have introduced selection bias. Sixth, myocardial perfusion territories were assigned according to the standardized 17-segment AHA model without systematic adjustment for individual coronary dominance patterns, which may have introduced some degree of vessel-level misclassification. Finally, while the sample size was larger than prior studies, subgroup analyses, particularly those involving vessel-level imaging and physiology, may remain underpowered for certain stratifications.

## Conclusions

This study identified distinct coronary phenotypes across vessels with normal function, CMD, and epicardial flow-limiting disease. CMD vessels were characterized by intermediate plaque burden, and pronounced positive remodeling, representing a unique structural–functional profile. The association between positive remodeling and preserved MRR underscores a potential link between epicardial morphology and microvascular function. Together, these findings highlight the complex interplay between microvascular and epicardial pathology and support further longitudinal studies to clarify their temporal relationships and prognostic implications.

## Supplementary Information

Below is the link to the electronic supplementary material.

Supplementary file1 (DOCX 29 KB)

## Data Availability

The data underlying this article are not publicly available due to study restrictions and data privacy considerations.
