# Supplementary material for: PET-based assessment of coronary microvascular dysfunction and its relation to plaque burden and vascular remodeling: integration with microvascular resistance reserve
Source: Eur J Nucl Med Mol Imaging. 2026 Jul 6;53(12):7573–83. doi: 10.1007/s00259-026-08054-3 (PMC13633150; doi:10.1007/s00259-026-08054-3)
Supplement: Supplementary file 1 [file 259_2026_8054_MOESM1_ESM.docx]

**Table S1. Prediction of MRR in non-ischemic vessels**

| Parameters | Univariable | | Multivariable | | |
| --- | --- | --- | --- | --- | --- |
|  | B (95%CI) | P-value | | B (95%CI) | P-value |
| Age, per 1 year increase | -0.01 (-0.04, 0.01) | 0.247 | |  |  |
| Male | -0.19 (-0.55, 0.17) | 0.305 | |  |  |
| BMI, per 1 kg/m^2^ increase | 0.01 (-0.04, 0.06) | 0.602 | |  |  |
| Diabetes mellitus | -0.05 (-0.51, 0.42) | 0.839 | |  |  |
| Hypertension | -0.07 (-0.43, 0.30) | 0.729 | |  |  |
| Hyperlipidemia | 0.06 (-0.34, 0.47) | 0.761 | |  |  |
| Smoking history | -0.15 (-0.51, 0.21) | 0.404 | |  |  |
| Family history of CAD | 0.07 (-0.29, 0.43) | 0.707 | |  |  |
|  |  |  | |  |  |
| **Symptoms** |  |  | |  |  |
| Typical angina | Reference |  | |  |  |
| Atypical angina | 0.22 (-0.29, 0.73) | 0.401 | |  |  |
| Aspecific chest pain | 0.11 (-0.32, 0.55) | 0.616 | |  |  |
|  |  |  | |  |  |
| **Imaging characteristics** |  |  | |  |  |
| Diameter stenosis, per 5% increase | -0.01 (-0.04, 0.02) | 0.436 | |  |  |
| Diameter stenosis ≥ 50% | -0.20 (-0.49, 0.09) | 0.174 | |  |  |
| Remodeling index, per 10% increase | -0.02 (-0.08, 0.03) | 0.448 | |  |  |
| Average lumen area, per 1 mm^2^ increase | 0.05 (-0.01, 0.11) | 0.101 | |  |  |
|  |  |  | |  |  |
| **Percentage atheroma volume, per 5% increase** |  |  | |  |  |
| Total plaque | -0.05 (-0.10, 0.01) | 0.022 | |  |  |
| Calcified plaque | -0.06 (-0.14, 0.02) | 0.119 | |  |  |
| Non-calcified plaque | -0.12 (-0.21, -0.04) | 0.006 | |  |  |

Abbreviations: BMI, body mass index; CAD, coronary artery disease; CI, confidence interval; CMD, coronary microvascular dysfunction

**Table S2. Prediction of MRR in epicardial flow-limiting vessels**

| Parameters | Univariable | | Multivariable | |
| --- | --- | --- | --- | --- |
|  | B (95%CI) | P-value | B (95%CI) | P-value |
| Age, per 1 year increase | -0.03 (-0.07,0.00) | 0.084 |  |  |
| Male | -0.65 (-1.84, 0.54) | 0.283 |  |  |
| BMI, per 1 kg/m^2^ increase | -0.08 (-0.18, 0.16) | 0.100 |  |  |
| Diabetes mellitus | -0.45 (-1.08, 0.18) | 0.164 |  |  |
| Hypertension | -0.80 (-1.36, -0.23) | 0.006 | -0.73 (-1.26, -0.21) | 0.007 |
| Hyperlipidemia | 0.17 (-0.44, 0.77) | 0.591 |  |  |
| Smoking history | -0.09 (-0.71, 0.52) | 0.764 |  |  |
| Family history of CAD | 0.07 (-0.53, 0.67) | 0.827 |  |  |
|  |  |  |  |  |
| **Symptoms** |  |  |  |  |
| Typical angina | Reference |  |  |  |
| Atypical angina | 0.22 (-0.46, 0.89) | 0.530 |  |  |
| Aspecific chest pain | 0.11 (-0.64, 0.85) | 0.782 |  |  |
|  |  |  |  |  |
| **Imaging characteristics** |  |  |  |  |
| Diameter stenosis, per 5% increase | -0.02 (-0.09, 0.05) | 0.585 |  |  |
| Diameter stenosis ≥ 50% | -0.14 (-0.88, 0.61) | 0.715 |  |  |
| Remodeling index, per 10% increase | -0.12(-0.23, -0.00) | 0.042 | -0.03 (-0.15, 0.09) | 0.630 |
| Average lumen area, per 1 mm^2^ increase | 0.05 (-0.18, 0.27) | 0.688 |  |  |
|  |  |  |  |  |
| **Percentage atheroma volume, per 5% increase** |  |  |  |  |
| Total plaque | -0.09 (-0.18, 0.04) | 0.062 |  |  |
| Calcified plaque | -0.07 (-0.18, 0.03) | 0.163 |  |  |
| Non-calcified plaque | -0.21 (-0.41, -0.01) | 0.044 | -0.17 (-0.40, 0.07) | 0.162 |

Abbreviations: BMI, body mass index; CAD, coronary artery disease; CI, confidence interval; CMD, coronary microvascular dysfunction

**Table S3. Multivariable analysis of MRR in CMD vessels**

| Parameters | Multivariable | | Multivariable | |
| --- | --- | --- | --- | --- |
|  | B (95%CI) | P-value | B (95%CI) | P-value |
| Remodeling index, per 10% increase | 0.09 (0.03, 0.16) | 0.006 | 0.11 (0.03, 0.18) | 0.004 |
| **Percentage atheroma volume, per 5% increase** |  |  |  |  |
| Total plaque | 0.05 (-0.02, 0.12) | 0.198 |  |  |
| Calcified plaque |  |  | 0.03 (-0.08, 0.14) | 0.568 |

**Table S4. Prediction of MRR in CMD vessels adjusting for vessel type**

| Parameters | Univariable | | Multivariable | |
| --- | --- | --- | --- | --- |
|  | B (95%CI) | P-value | B (95%CI) | P-value |
| **Vessel type** |  |  |  |  |
| RCA | Ref. | Ref. | Ref. | Ref. |
| LAD | 0.12 (-0.14, 0.84) | 0.360 | 0.03 (-0.16, 0.23) | 0.749 |
| LCX | 0.14 (-0.05, 0.33) | 0.139 | 0.19 (-0.01, 0.39) | 0.063 |
| Remodeling index, per 10% increase | 0.12 (0.07, 0.17) | <0.001 | 0.12 (0.06, 0.17) | <0.001 |
| PAV-NCP | 0.18 (0.03, 0.33) | 0.021 | 0.08 (-0.05, 0.20) | 0.231 |

LAD, left anterior descending artery; LCX, left circumflex artery; NCP, noncalcified plaque; PAV, Percentage atheroma volume; RCA, right coronary artery
